# Comment on “Total Magnesium Intake and Risk of Frailty in Older Women” by Struijk et al.—The Authors' Reply

**DOI:** 10.1002/jcsm.13653

**Published:** 2024-12-03

**Authors:** Ellen A. Struijk, Teresa T. Fung, Heike A. Bischoff‐Ferrari, Walter C. Willett, Esther Lopez‐Garcia

**Affiliations:** ^1^ Department of Preventive Medicine and Public Health, School of Medicine Universidad Autónoma de Madrid‐IdiPaz Madrid Spain; ^2^ CIBERESP (CIBER of Epidemiology and Public Health) Madrid Spain; ^3^ Department of Nutrition Simmons University Boston Massachusetts USA; ^4^ Department of Nutrition Harvard T.H. Chan School of Public Health Boston Massachusetts USA; ^5^ Department of Geriatrics and Aging Research University Hospital Zürich and University of Zürich Zürich Switzerland; ^6^ Centre on Aging and Mobility University Hospital Zürich and Waid City Hospital Zürich Switzerland; ^7^ Channing Division of Network Medicine, Department of Medicine Brigham and Women's Hospital and Harvard Medical School Boston Massachusetts USA; ^8^ IMDEA/Food Institute, CEI UAM + CSIC Madrid Spain

Dear Editor,

We would like to thank you for the opportunity to respond to the issues raised in the letter by Drs Guo, Lan, Zhou and Liu [1]. The authors raise the concern that the long follow‐up of our cohort study has affected the robustness of the study results. The data used from the Nurses' Health Study have the advantage of repeated dietary measurements over a long study period. Therefore, the cumulative average of dietary magnesium intake was assessed in association with frailty incidence since this best reflects long‐term intake [2]. However, due to the strong increase in supplement intake over the years, we used a different approach for the supplemental magnesium intake and considered the most recent supplemental magnesium intake before frailty onset or the end of follow‐up. Additionally, when no specific supplement brand was given, the supplemental magnesium intake was estimated as well as possible based on the most frequently used supplement on the market in the year the food frequency questionnaire was returned.

The authors of the letter are right that supplement users may be more likely to already have a deteriorated functional status. We have addressed this by repeating the analysis excluding women who were prefrail and by excluding women with cancer, diabetes, or heart disease at baseline; the association between supplemental magnesium and frailty remained non‐significant.

We agree that social workers are important players in supporting the needs of older adults and more research is needed to understand how supplement use can improve health among older adults. Older adults for whom healthy eating can be a challenge due to dental problems, chewing difficulties, high costs of healthy foods or loss of appetite, a multivitamin supplement including magnesium may be of benefit to reach an adequate nutrient intake [3].

The main message of our study is that, for most older adults, an optimal palatable diet pattern that provides enough minerals, as well as an optimal macronutrient composition, is likely to help prevent the development of geriatric syndromes.
